# Flood-Induced Changes in Soil Microbial Functions as Modified by Plant Diversity

**DOI:** 10.1371/journal.pone.0166349

**Published:** 2016-11-21

**Authors:** Odette González Macé, Katja Steinauer, Alexandre Jousset, Nico Eisenhauer, Stefan Scheu

**Affiliations:** 1 J.F. Blumenbach Institute of Zoology and Anthropology, University of Göttingen, Berliner Str. 28, 37073, Göttingen, Germany; 2 German Centre for Integrative Biodiversity Research (iDiv) Halle-Jena-Leipzig, Deutscher Platz 5e, 04103, Leipzig, Germany; 3 Institute of Biology, Leipzig University, Johannisallee 21, 04103, Leipzig, Germany; 4 Institute of Environmental Biology, Utrecht University, Padualaan 8, 3584 CH, Utrecht, The Netherlands; Pacific Northwest National Laboratory, UNITED STATES

## Abstract

Flooding frequency is predicted to increase during the next decades, calling for a better understanding of impacts on terrestrial ecosystems and for developing strategies to mitigate potential damage. Plant diversity is expected to buffer flooding effects by providing a broad range of species’ responses. Here we report on the response of soil processes to a severe summer flood in 2013, which affected major parts of central Europe. We compared soil microbial respiration, biomass, nutrient limitation and enzyme activity in a grassland biodiversity experiment in Germany before flooding, one week and three months after the flood. Microbial biomass was reduced in the severely flooded plots at high, but not at low plant functional group richness. Flooding alleviated microbial nitrogen limitation, presumably due the input of nutrient-rich sediments. Further, the activity of soil enzymes including 1,4-β-N-acetylglucosaminidase, phenol oxidase and peroxidase increased with flooding severity, suggesting increased chitin and lignin degradation as a consequence of the input of detritus in sediments. Flooding effects were enhanced at higher plant diversity, indicating that plant diversity temporarily reduces stability of soil processes during flooding. The long-term impacts, however, remain unknown and deserve further investigation.

## Introduction

Climate change has increased the frequency and magnitude of extreme weather events such as wildfires and floods [1,2]. Floods affect soil structure and fertility, reducing nutrient availability and initiating primary succession processes in the case of strong disturbances [2]. However, moderate floods also may beneficially affect nutrient cycling and increase habitat heterogeneity thereby fostering biodiversity [2]. Biodiversity itself can influence the way ecosystems respond to disturbances such as flooding. For example, plant diversity has been shown to increase the resistance of community functions to wet (as well as dry) conditions [3]. However, results from a recent flood which hit the Jena Experiment, a large scale field experiment exploring plant diversity-ecosystem functioning relationships [4], suggest that stability actually decreases at higher plant diversity due to greater biomass production at low flooding severity, and low biomass production at high flooding severity [5]. There is little knowledge, however, about how soil processes are affected by interactions between plant diversity and disturbances like flooding [6].

Soil microorganisms are vital for the functioning and long-term sustainability of ecosystems [7]. They play key roles in organic matter decomposition and nutrient cycling and thereby for plant growth. Plant diversity alters microclimatic conditions as well as the quantity and quality of litter and root resources [8–10], thereby controlling the composition and functioning of soil microbial communities [11,12]. Indeed, previous studies in the framework of the Jena Experiment suggest that microbial respiration and biomass increase with plant species richness [9,10,13]. Microorganisms at this site are limited primarily by nitrogen, with the limitation being more pronounced at low plant diversity due to lower plant N capture than under high plant diversity [9].

Floods provide rich and readily decomposable nutrients and alter nitrogen dynamics, which influence nutrient availability and alter plant growth [14,15]. In parallel, soil inundation also results in oxygen depletion, fostering anaerobic conditions and microorganisms able to survive these conditions [16]. Such changes in soil abiotic conditions can alter soil microbial community composition [6,17]. During the flooding of the Jena Experiment, plant diversity did not attenuate flooding effects on soil microbial communities composition [6], even though plant community functions were altered after the flood [5].

One of the major indicators of soil microbial functioning is enzyme activity [18]. Soil enzymes play an essential role in organic matter decomposition and nutrient cycling [18]. Although soil enzymes mainly are synthesized by soil microorganisms, some are also produced by plant roots [19]. Enzymes degrade polymers of microbial and plant origin, such as cellulose, chitin and lignin, into smaller units to be used for microbial growth or plant uptake [20]. Enzyme activity is regulated by the demand for substrate as well as by substrate availability [21]. Previous studies reported that the activity of extracellular enzymes in soil is enhanced by increasing plant diversity [22–24]. Flooding also is likely to affect soil enzyme activities by changing nutrient availability and oxygen concentrations as well as due to shifting microbial community composition [25].

In the present study we focus on changes in microbial respiration, biomass and nutrient status as well as enzyme activity associated with the 2013 summer flood which hit the Jena Experiment. We expected an immediate decrease in basal respiration, microbial biomass and enzyme activities due to anoxic conditions, particularly so at high flooding severity [5]. Further, we expected effects of flooding on microbial biomass and enzyme activities to be more pronounced in higher plant diversity plots due to high oxygen consumption by roots in more diverse plant communities with higher root biomass [26–28]. Also, we expected the flood to reduce microbial nutrient limitation due to the input of nutrient-rich sediments and the enhanced availability of dead organic material with the reduction being most pronounced at high plant diversity due to more severe nutrient limitation via more efficient plant N capture at high plant diversity. With time we expected plant diversity to foster recovery of microbial biomass and enzyme activities after the flood due to enhancing internal nutrient cycling and higher input of root-derived residues.

## Material and Methods

### Experimental design

The experiment was located in a semi-natural temperate grassland on a Eutric Fluvisol [29] in the floodplain of the river Saale close to the city of Jena (Thuringia, Germany, 50° 55′ N, 11° 35′ E, 130 m a.s.l.). Mean annual air temperature is 9.9°C and mean annual precipitation is 610 mm (1980–2010) [30]. The study site had been used as arable field for over 40 years before the experiment was established in 2002. The experiment comprises 80 plots of 5 x 6 m arranged in 4 blocks to control for changes in soil texture with distance from the river. A gradient of plant species richness (1, 2, 4 8, 16 and 60) and plant functional group richness (1, 2, 3 and 4) was established comprising typical plant Central European hay meadow species. Species are grouped according to the morphological, phenological and physiological traits into grasses (16 species), small herbs (12 species), tall herbs (20 species) and legumes (12 species) (for details see [4]). The established grassland is mown twice a year and weeded three times per year [4].

### Flooding

Rainfall in May 2013 in Jena was approximatively 150 mm (>25% of annual precipitation at the site) and the experimental field was flooded for 24 days (30 May to 24 June). Flooding caused anaerobic soil conditions, as shown by redox potentials ranging from -121 to 193 mV in the soil of some plots [5]. Water coverage was measured for each plot every day from 31 May to 24 June and ascribed to 5 levels: 0, 25, 50, 75 and 100%. Flooding severity was evaluated using a continuous flooding index (sum of the percentage water coverage per day over the flooding period) [5].

### Microorganisms

Soil samples were taken from each plot before the flood (16^th^ May 2013) and twice after the flood (1^th^ July and 17^th^ September 2013), i.e. at maximum and at late plant growth. Samples were based on composite samples of three soil cores of a diameter of 5 cm to a depth of 5 cm. The samples were homogenized, sieved (2 mm) and stored at 5°C until further analysis.

Basal respiration (BR) and substrate-induced respiration (SIR; [31]) were measured using an O_2_ microcompensation apparatus [32]. Respiration was measured at 22°C with readings taken at hourly intervals. BR (μL O_2_ g^-1^ soil dry weight h^-1^) was calculated as mean O_2_ consumption rates from 14 to 24 h after attachment of the samples to the respiration apparatus. SIR was measured after addition of D-glucose saturating the catabolic enzymes of the microorganisms (4 mg g^-1^ dry weight solved in 400 μL deionized water; cf. [9]). The lowest three readings within the first 10 h were averaged as the maximum initial respiratory response (MIRR; μL O_2_ g^-1^ soil dry weight h^-1^) and microbial biomass (C_mic_; μg C g^-1^ soil dry weight) was calculated as 38 × MIRR [33] (S1 Table).

Microbial nutrient limitations were evaluated by measuring the respiratory response after addition of D-glucose and nutrients as aqueous solution (400 μL g^-1^ soil dry weight) to fresh soil equivalent to 3.5 g dry mass. Glucose (C), glucose and nitrogen (CN; N as (NH_4_) SO_4_), glucose and phosphorus (CP; P as K_2_HPO_4_), and glucose, nitrogen and phosphorus (CNP) were added in a mass ratio of C: N: P of 10:2:1 [34]. Respiration rates between the lowest (usually 3–6 h after substrate addition) and highest reading were taken to calculate microbial growth [35]. Data were ln-transformed and the slope determined by linear regression (S2 Table).

### Enzyme activity

We measured enzymes involved in carbon, nitrogen, phosphorus and sulfur cycling. Reaction products of the lignin degrading enzymes phenol oxidase and peroxidase (C turnover) were measured spectrophotometrically using 5 mM L-3,4-dihydroxy-phenylalanine as substrate. A sample suspension was prepared from 1 g soil and 125 ml of 50 mM TRIS buffer (pH 7.9) and homogenized using an ultrasonic bath for 1 min. According to the pH value of the soil samples, pH of 7.9 was chosen for the assays. Absorbance of phenol oxidase and peroxidase was determined using 96-wells plates with four replicates of each soil sample, four blanks and six replicates of negative control [36]. The activities of phenol oxidase and peroxidase were measured at 450 nm after an incubation period of 14 h in darkness at 20°C using an absorbance microplate reader (FLUOstar Omega, BMG Labtech, Ortenberg, Germany). All soil enzyme activities were expressed in nanomoles per gram of dry soil per hour (nmol g^-1^ soil dry weight h^-1^).

Activities of 1,4-β-N-acetylglucosaminidase, β-glucoronidase, galactosidase (all C turnover), phosphatase (P turnover), sulfatase (S turnover) and two proteases (cleaving at arginine and tyrosine residues, respectively; N turnover) were assessed with a 96-well microplate fluorogenic assay described by [37]. Briefly, 0.1 g of fresh soil were dissolved in 10 ml sterile water and homogenized by shaking in a horizontal shaker at 200 min^-1^ for 30 min. The soil slurry was mixed with 40 mM of the appropriate substrate in 0.1 MES buffer (pH 6.1) and fluorescence increase recorded continuously in a Tecan M200 plate reader (ex/em 365/460, gain 60). Enzyme activity was defined as Methylumbeliferone (MUB)-equivalent released and gram of soil per minute. In order to account for quenching fluorescence signal by soil particles, a serial dilution of MUB was set up in a reference soil slurry (S3 Table).

### Statistical analyses

Data were inspected for normality and homoscedasticity of errors using Shapiro—Wilk normality test (P > 0.05) and Fligner—Killeen test (P > 0.05). The 60 species mixtures were excluded from the statistical analysis due to an insufficient number of replicates (four replicates at the field site, all being differentially affected by the flood; [5]). The difference of respective values at the two sampling dates after the flood (July or September) and before the flood (May) are used as dependent variables. Thereby, we controlled for possible confounding of flooding with other site-specific variables. Plant species richness was log-transformed to linearize the saturating relationship between plant diversity and microbial properties [38].

Linear models were used to analyze the effects of block (categorical variable, 4 blocks), flooding index (continuous variable, from 1 to 23), log plant species richness (continuous variable, from 1 to 16), functional group richness (continuous variable, from 1 to 4) and the presence/absence of legumes, grasses, tall herbs and short herbs (categorical variable) on the differences in basal respiration, microbial biomass, microbial growth after addition of C, CP, CN and CNP and enzyme activity (phenol oxidase, peroxidase, 1,4-β-N-acetylglucosaminidase, β-glucoronidase, galactosidase, phosphatase, sulfatase, arg-protease and tyr-protease). Interactions between plant diversity (plant species richness and plant functional group richness) and the presence/absence of legumes, grasses, tall herbs and small herbs were checked against flooding index. The full model with the lowest Akaike Information Criterion (AIC) was selected as the best starting model [39,40]. This model was simplified in a stepwise manner by dropping non-significant variables. Although the experimental design was set up as orthogonally as possible, there is collinearity between functional group richness of plants and the presence/absence of individual functional groups [4], which we quantified using the inflation factor (VIF) from the car package [41]. The analysis suggested to exclude functional group richness if there are two or more functional groups in the model (VIF ~ 4). Therefore, functional group richness was added after model simplification and was only included in the final model if it improved the model significantly (principle of Occam’s Razor, p-value < 0.05). F-values given in text and tables refer to models in which the respective factor was fitted first [42]. Block and flooding index were fitted first followed by plant species richness; thereafter presence/absence of grasses, legumes, tall herbs and short herbs were fitted. Statistical analyses were performed using R 3.2.1 [43].

## Results

### Immediate responses to the flood (July versus May)

**Fig 1:**
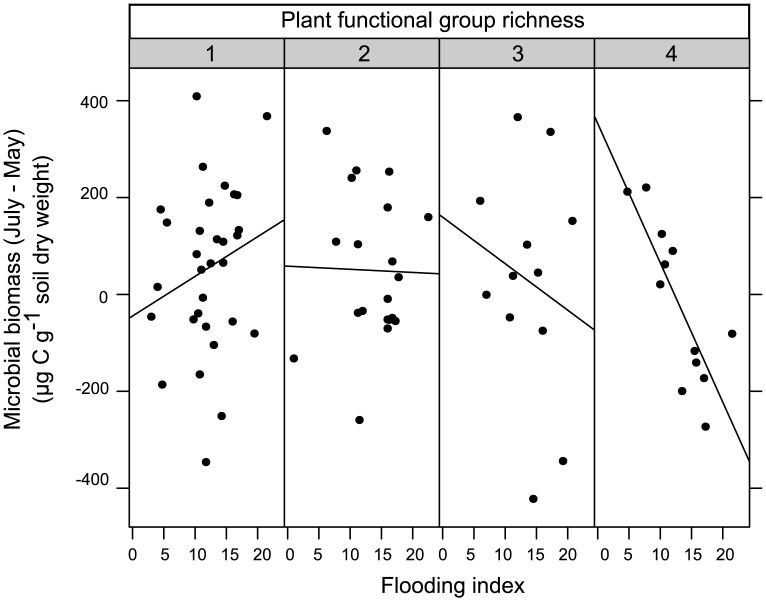
Variation in microbial biomass depending on flooding index and plant functional group diversity. Effects of flooding index and plant functional groups richness (1, 2, 3 and 4) on microbial biomass changes (μg C g^-1^ soil dry weight) between May and July (p < 0.01). For the purposes of display only, plant functional group richness are split into 4 levels (1 to 4).

**Fig 2:**
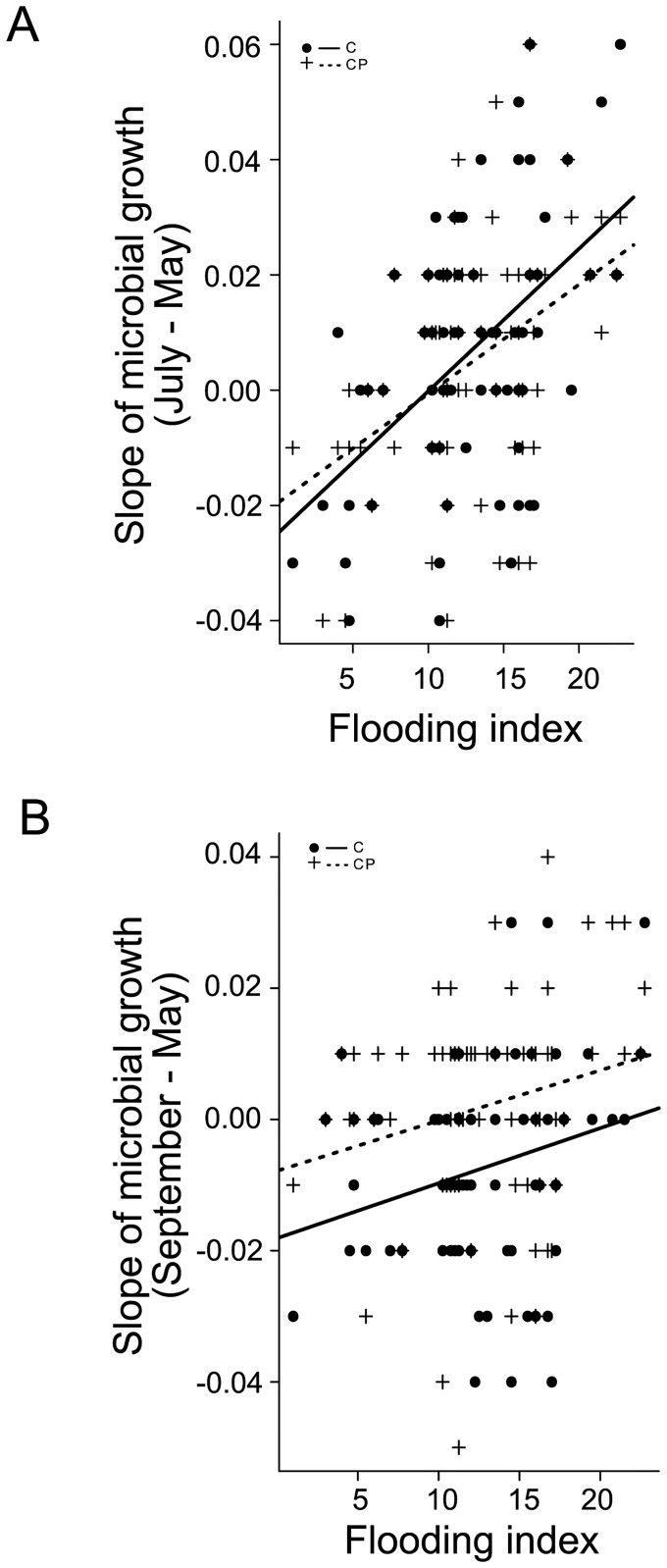
Effects of flooding on C- and CP-supplemented microorganisms. Variation in slopes of regression lines of microbial growth of carbon-supplemented (C; dots) and carbon and phosphorus-supplemented (CP;cross) microorganisms between (A) May and July (R_C_^2^ = 0.19, R_CP_^2^ = 0.20; p < 0.01 for both), and (B) May and September (R_C_^2^ = 0.04, R_CP_^2^ = 0.05; p < 0.05 for both) depending on flooding index.

**Fig 3:**
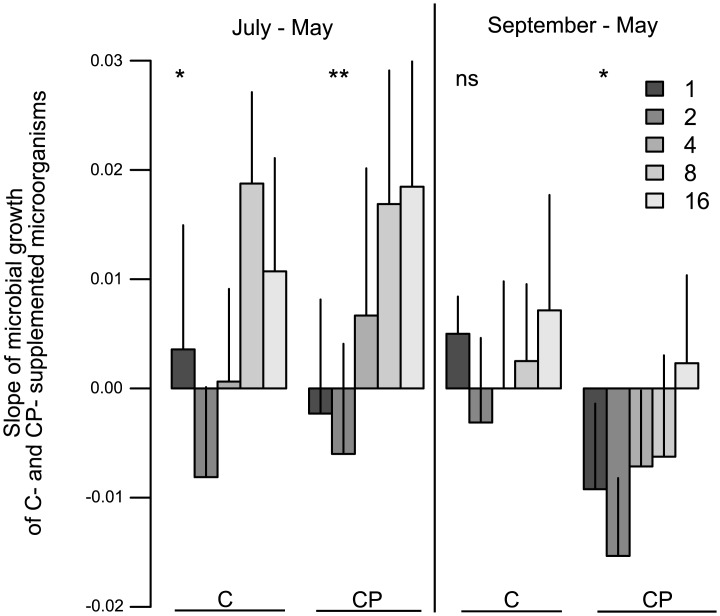
Variation in C- and CP-supplemented microorganisms depending on plant species richness. Effects of plant species richness (1, 2, 4, 8 and 16) on slope changes of microbial growth of carbon-supplemented (C) and carbon and phosphorus-supplemented (CP) microorganisms between July and May and September and May. Asterisks indicate significant differences (**p < 0.01, *p < 0.05, ns = not significant). Values are means ± SE.

As indicated by measurements immediately before (May) and after the flood (July) basal respiration was not significantly affected by flooding index; rather, only the presence of legumes increased basal respiration (Table 1). In contrast, microbial biomass significantly changed with flooding index, but the effect varied with plant functional group richness, with a slight increase at low plant functional group richness and a strong decrease in more severely flooded plots of high plant functional group richness (Fig 1,Table 1). Further, differences of microbial growth of C- and CP-supplemented microorganisms increased with increasing flooding index (Fig 2A, Table 1) and with increasing plant species richness (Fig 3, Table 1). Moreover, legume presence significantly reduced changes in microbial growth of CP-supplemented microorganisms (Table 1). In contrast, changes in microbial growth of CN- and CNP-supplemented microorganisms did not vary significantly with flooding and plant community properties.

**Table 1:** Flooding and plant community effects on soil microbial functions.

May–July												
	BR		C_mic_		C		CN		CP		CNP	
	df	F	df	F	df	F	df	F	df	F	df	F
Block	3,51	**6.09**		-		-		-		-	3,64	*3*.*46*
FI		-	1,71	0.55	1,73	**18.58**		-	1,68	**23.30**		-
FG		-	1,71	1.44		-		-		-		-
SR		-		-	1,73	**6.08**		-	1,68	**8.90**		-
Leg	1,51	**10.90**		-		-		-	1,68	**12.20**		-
Interactions												
FI × FG		-	1,71	**8.50**		-		-		-		-
May–September												
	BR		C_mic_		C		CN		CP		CNP	
	df	F	df	F	df	F	df	F	df	F	df	F
Block	3,52	**6.44**		-	3,73	*3*.*29*		-		-	3,62	**3.19**
FI		-		-	1,73	**5.11**		-	1,68	**4.58**		-
SR		-		-		-		-	1,68	**4.40**		-
Sh		-	1,74	**8.13**		-	1,72	*3*.*67*		-		-
Th		-		-		-	1,72	**5.85**		-		-

F-value table of linear models on the effect of block, flooding index (FI), plant functional group richness (FG), plant species richness (SR), presence of legumes (Leg), small herbs (Sh) and tall herbs (Th) on changes in basal respiration (BR), microbial biomass (C_mic_), slope in microbial growth after addition of carbon (glucose, C), carbon and nitrogen (CN), carbon and phosphorus (CP) and carbon, nitrogen and phosphorus (CNP) between May and July, and May and September. Significant effects (p < 0.05) are highlighted in bold and marginally significant results (p < 0.10) are given in italics.

**Fig 4:**
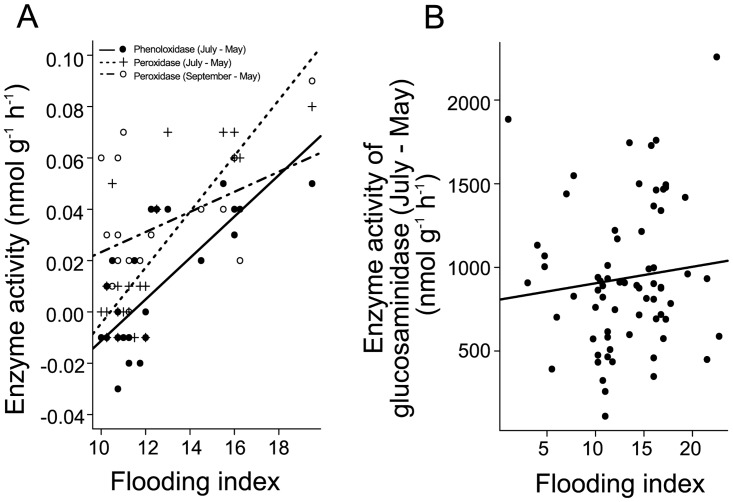
Changes in enzyme activities as affected by flooding index. (A) Variation in phenol oxidase between May and July (black dots, p < 0.01, R^2^ = 0.58), peroxidase between May and July (black cross, p < 0.01, R^2^ = 0.71) and peroxidase between May and September (white dots, p < 0.05, R^2^ = 0.22) depending on flooding index, and (B) variation in 1,4-β-N-acetylglucosaminidase activity between May and July influenced by flooding index (p < 0.05, R^2^ = 0.02).

**Fig 5:**
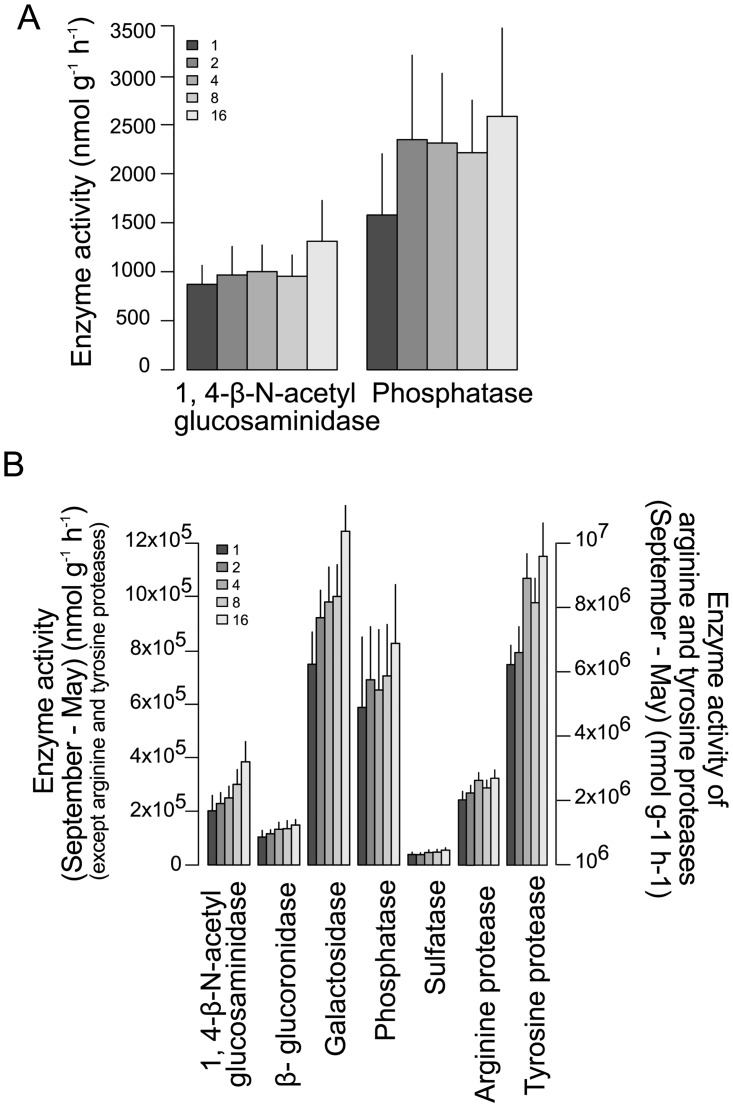
Significant changes in enzyme activities as affected by plant species richness. (A) Variation in 1,4-β-N-acetylglucosaminidase and phosphatase activity between May and July affected by plant species richness (1, 2, 4, 8 and 16; p < 0.01), and (B) variation in glucosaminidase, β-glucoronidase, galactosidase, phosphatase, sulfatase, arginine protease and tyrosine protease activity between May and September depending on plant species richness (p < 0.01). Values are means ± SE.

Differences of the activities of phenol oxidase, peroxidase and 1,4-β-N-acetylglucosaminidase were more pronounced in more heavily flooded plots (Fig 4A and 4B, Table 2). Further, plant species richness significantly enhanced changes in 1,4-β-N-acetylglucosaminidase and phosphatase activity (Fig 5A, Table 2). The presence of legumes increased changes in 1,4-β-N-acetylglucosaminidase, galactosidase and sulfatase activities, while the presence of grasses increased changes in phosphatase activities (Table 2). Small herbs presence only reduced changes in tyrosine protease activity.

**Table 2:** Flooding and plant community effects on soil enzymes.

May–July																		
Cycle	Carbon										Phosphorus		Sulphur		Nitrogen			
Enzymes	Phenol oxidase		Peroxidase		1,4-β- N -acetyl glucosaminidase		β-glucoronidase		Galactosidase		Phosphatase		Sulfatase		Arginine protease		Tyrosin protease	
Factors	df	F	df	F	df	F	df	F	df	F	df	F	df	F	df	F	df	F
Block	1,26	**134.26**	1,24	**221.36**	3,62	**12.18**	3,65	**9.66**	3,69	**8.48**	3,66	**30.18**	3,63	**27.05**	3,71	*2*.*39*		-
FI	1,26	**7.01**	1,24	**23.66**	1,62	**4.47**		-		-		-		-		-		-
SR		-		-	1,62	**13.95**		-		-	1,66	**7.45**		-		-		-
Gr		-		-		-		-		-	1,66	**4.46**		-		-		-
Leg		-		-	1,62	**24.10**		-	1,69	**7.96**		-	1,63	**6.33**		-		-
Sh		-		-		-		-		-		-		-		-	1,73	**8.39**
May–September																		
Cycle	Carbon										Phosphorus		Sulphur		Nitrogen			
Enzymes	Phenol oxidase		Peroxidase		1,4-β- N -acetyl glucosaminidase		β-glucoronidase		Galactosidase		Phosphatase		Sulfatase		Arginine protease		Tyrosin protease	
Factors	df	F	df	F	df	F	df	F	df	F	df	F	df	F	df	F	df	F
Block		-		-	3,66	**4.80**	3,63	**7.13**	3,67	**6.10**	3,69	**92.30**	3,67	**14.99**	3,68	**4.87**	3,68	**3.91**
FI		-	1,24	**6.97**		-		-		-		-		-		-		-
SR		-		-	1,66	**26.55**	1,63	**12.93**	1,67	**42.99**	1,69	**10.74**	1,67	**15.99**	1,68	**48.37**	1,68	**14.37**
Gr	1,20	**20.86**		-		-		-		-		-		-	1,68	**4.17**		-
Leg	1,20	**5.22**		-		-		-		-		-		-		-		-
Sh		-		-		-		-	1,67	**4.32**		-		-	1,68	**5.00**	1,68	**4.69**

F-value table of linear models on the effect of block, flooding index (FI), plant species richness (SR), presence of grasses (Gr), legumes (Leg) and small herbs (Sh) on changes in the activity of phenol oxidase, peroxidase, 1,4-β-N-acetylglucosaminidase, β-glucoronidase, galactosidase, phosphatase, sulfatase, arginine protease and tyrosine protease between May and July, and May and September. Significant effects (P < 0.05) are highlighted in bold and marginally significant effects (P<0.10) are given in italics.

### Medium-term responses to the flood (September versus May)

Three months after flooding (September) changes in microbial growth of C- and CP-supplemented microorganisms were more pronounced in flooded plots (Fig 2B, Table 1). Further, changes in microbial growth of CP-supplemented microorganisms were stronger with plant species richness and these changes were positive at highest species richness (Fig 3). In addition, changes in microbial biomass were more pronounced in the presence of small herbs (Table 1). Moreover, changes in microbial growth of CN-supplemented microorganisms were stronger in the presence of tall herbs (Table 1).

As immediately after the flood, changes in peroxidase activity were significantly affected by flooding index in September (Fig 4A). Further, changes in the activities of each of the enzymes measured were significantly positively affected by plant species richness except for peroxidase and phenol oxidase (Fig 5B). Further, changes in phenol oxidase and arginine protease activity increased with the presence of grasses, while changes in arginine and tyrosine protease and galactosidase increased significantly with the presence of small herbs. Moreover, changes in phenol oxidase activity were more pronounced in presence of legumes.

## Discussion

Contrary to our expectation, basal respiration was not affected by flooding. This is surprising as the soils were anoxic in heavily flooded plots [5], which it is expected to infer proliferation of anaerobic microorganisms. Rather, the result suggests that aerobic microorganisms recovered quickly after flooding. As the plants also recovered fast, plant root exudation may have increased the carbon input intoo the soil, and, consequently, counteracted the negative effect of the flood on microbial respiration [17]. Supporting this conclusion, microbial biomass also responded little to flooding, being only reduced in plots with high plant functional group diversity. Presumably, high plant functional group diversity aggravated oxygen limitation in flooded plots due to higher root biomass and root respiration as well as associated increased microbial biomass and respiration [9,13,16,44,45]. Flooding may favor fast-growing microorganisms such as bacteria, which can readily use easily available substrates and by growing fast compensating short-term detrimental effects [6].

As we hypothesized, limitation of microorganisms by N was reduced, likely due to an increase in soil nitrate concentrations in flooded plots. In contrast, limitation by P and C was increased after flooding with the reduction being most pronounced in plots most severely affected by flooding, likely due to dead organic material and sediments deposited by the flood [5]. Of the studied enzyme activities, only those of phenol oxidase, peroxidase and 1,4-β-N-acetylglucosaminidase increased with increasing flood severity, suggesting that flooding fostered chitin and lignin degradation, presumably due to the input of additional dead organic material.

Flooding effects on soil processes were more severe at high plant diversity supporting earlier conclusions that plant diversity may compromise grassland stability [5]. In high diversity plots, microorganisms are less nutrient-limited as compared to low diversity plots [9], presumably due to the input of resources of higher quality at higher plant diversity. Before the flood, microorganisms were limited primarily by the availability of N. Due to flooding, microorganisms benefited from the input of N-rich sediments in particular at high plant diversity, presumably due to complex plant structure increasing sedimentation. Moreover, mortality of some soil animals was very high during the flood (N. Eisenhauer, personal observation), which have higher densities at high plant diversity [46] and which might have also increased N availability for soil microorganisms through the turnover of dead bodies. As a consequence, however, limitation of P and C was increased immediately after the flood.

In general, plant diversity increases both above- and belowground plant biomass [28,45], as well as substrate availability, microbial biomass and, as a consequence, enzyme activity [47]. The increase in the activity of phosphatase and 1,4-β-N-acetylglucosaminidase with plant species richness in July probably reflects these interrelationships. The stronger effect of plant diversity on almost all enzyme activities in September (except for peroxidase and phenol oxidase) may be related to seasonality. In September, at the end of the vegetation period when plants start to die back and increased amounts of plant residues enter the soil, microbial biomass [48] and enzyme activity increases.

Some of the effects of flooding might have been related to characteristics of specific functional groups of microorganisms. The increase in basal respiration, 1,4-β-N-acetylglucosaminidase, galactosidase and sulfatase activity in presence of legumes shortly after the flood likely resulted from increased microbial growth due to high quality inputs (low C/N ratio) from legume litter, since legumes were most affected by severe flooding [5,49,50]. Supporting this conclusion, limitation of microorganisms by P is aggravated, whereas that by N is alleviated, in presence of legumes [9,50,51], and this was true in July in the present study. Three months after the flood, however, limitation by P exceeded that before the flood with the limitation being independent of the presence of legumes. The lack of legume effect in September presumably reflects that plant growth and N_2_ fixation by rhizobia in the rhizosphere of legumes associated with high P demand had not recovered yet after the flood [52].

In contrast to legumes, small herbs are generally shallow-rooting with most roots being concentrated in the uppermost soil layer [53]. The presence of small herbs increased microbial biomass, galactosidase, and arginine and tyrosine protease activity three months after the flood, presumably, due to increased rhizodeposition in the topsoil. Furthermore, the presence of grasses slightly increased the activity of phosphatase in July, probably as a response to the P limitation right after the flood.

## Conclusion

Flooding was associated by the input of sediments rich in nutrients. As a consequence, N limitation decreased with flooding severity. Also, the activity of enzymes degrading recalcitrant compounds such as lignin and chitin increased with flooding. Notably, these effects were more pronounced at high plant diversity and varied with plant functional group identity. Shortly after the flood, effects of legumes predominated, presumably due to high P demands of legume-associated rhizobia. In contrast, three months after the flood the effect of small herbs were most pronounced suggesting increased rhizodeposition by small herbs into the topsoil. Generally, effects of flooding were more pronounced shortly after the flood than after three months suggesting that soil microorganisms and their functioning recovered quickly resulting in nutrient limitations resembling those before the flood. Notably, even immediately after the summer flood the biomass and activity of soil microorganisms were only moderately affected despite anoxic soil conditions. Overall, the results point to high resistance and resilience of soil microbial communities in grassland with plant diversity compromising ecosystem stability. However, future studies should investigate long-term compositional changes on soil communities as well as plant community effects on soil process responses to disturbances.

## Supporting Information

S1 Table Basal respiration and microbial biomass dataset belonging to the samples taken in May July and September. (PDF)

S2 Table Dataset of slopes of respiration rates after addition of Glucose (C), glucose and nitrogen (CN; N as (NH_4_) SO_4_), glucose and phosphorus (CP; P as K_2_HPO_4_), and glucose, nitrogen and phosphorus (CNP) from May July and September samples. (PDF)

S3 Table Enzymatic activity of 1,4-β-N-acetylglucosaminidase, β-glucoronidase, galactosidase, phosphatase, sulfatase, peroxidase, phenol oxidase and two proteases (cleaving at arginine and tyrosine residues, respectively) belonging to the samples taken in May July and September. (PDF)
