# Supplementary material for: Flood-Induced Changes in Soil Microbial Functions as Modified by Plant Diversity
Source: PLoS One. 2016 Nov 21;11(11):e0166349. doi: 10.1371/journal.pone.0166349 (PMC5117659; doi:10.1371/journal.pone.0166349)
Supplement: S1 Table — (PDF) [file pone.0166349.s001.pdf]

**Dataset**	Basal respiration	Microbial biomass (Cmic)
Type	raw	raw
Unit	[µg O2*h-1*g soil dw-1]	[µg Cmic*g soil dw-1]
Date(s)	2013-05-16	
**Plot**		
B1 A01	3.77	1249.94
B1 A02	2.77	946.85
B1 A03	2.63	957.69
B1 A04	2.51	2533.83
B1 A05	1.39	640.22
B1 A06	2.73	995.90
B1 A07	1.61	670.80
B1 A08	1.36	614.54
B1 A13	2.19	719.10
B1 A14	3.28	914.96
B1 A16	1.63	659.36
B1 A17	1.70	754.97
B1 A17	1.70	752.29
B1 A18	2.49	940.93
B1 A19	3.32	849.41
B1 A20	4.00	1353.31
B1 A21	3.86	1113.85
B1 A22	3.79	1216.21
B2 A01	3.13	939.97
B2 A02	3.61	935.41
B2 A03	4.73	1403.95
B2 A04	2.40	757.02
B2 A05		859.39
B2 A06	2.94	1082.46
B2 A08	2.55	1181.74
B2 A09	3.14	1491.17
B2 A10	3.96	1258.89
B2 A12	7.31	2192.97
B2 A13	4.90	1694.04
B2 A14	8.07	2141.57
B2 A15	4.22	2072.05
B2 A17	6.80	2642.05
B2 A18	7.29	2673.18
B2 A19	6.87	2526.55
B2 A20	6.41	2368.96
B2 A21	6.88	2705.95
B2 A22	9.13	2540.41
B3 A12	4.72	2541.91
B3 A14	8.46	2473.18
B3 A19	6.81	2205.52
B3 A21	4.02	1987.85
B3 A22		2592.49
B3 A23	8.21	
B3 A24	8.30	2167.28
B3 A01	2.86	883.12

B3 A02	2.00	863.41
B3 A03	2.47	1153.03
B3 A04	3.61	1352.03
B3 A05	4.01	1437.80
B3 A06		1245.18
B3 A07	2.93	1263.14
B3 A09	3.84	1081.85
B3 A11	4.14	1032.85
B4 A01	4.46	1306.86
B4 A02	3.79	1421.48
B4 A04	3.54	1282.88
B4 A05		1468.17
B4 A06	3.61	1354.19
B4 A08	3.19	977.00
B4 A09	2.06	923.26
B4 A10	2.78	1084.87
B4 A11	3.50	1010.95
B4 A12	3.25	910.34
B4 A13	1.58	769.25
B4 A14	2.42	951.93
B4 A15	2.11	969.61
B4 A16	2.12	976.06
B4 A17	1.99	954.03
B4 A18	3.87	1425.70
B4 A20	2.79	1105.10
B4 A21	2.47	983.47
B4 A22	3.37	1054.50
Date(s)	2013-07-01	
**Plot**		
B1A01	4.65	1179.53
B1A02	5.53	1238.99
B1A03	5.88	1265.81
B1A04	5.28	1031.49
B1A05	3.24	773.04
B1A06	4.20	1272.90
B1A07	3.33	1019.22
B1A08	3.54	952.02
B1A11	4.33	1200.52
B1A12	3.12	886.53
B1A13	5.34	849.68
B1A14	5.29	1210.64
B1A15		3914.54
B1A16	3.41	949.00
B1A17	3.21	827.14
B1A18	2.30	659.34
B1A19	3.74	879.94
B1A20	3.83	1175.92
B1A21	3.96	4078.45
B1A22	5.07	1381.36
B2A01	3.42	1080.82

B2A02	4.72	1071.96
B2A03	4.48	1147.63
B2A04		4247.13
B2A05	3.75	918.22
B2A06	4.11	
B2A08	2.34	947.39
B2A09	3.43	1184.80
B2A10	3.71	1254.89
B2A12	3.67	1241.56
B2A13	2.82	798.22
B2A14		4436.30
B2A15	3.85	790.26
B2A16	5.69	1444.90
B2A17	5.21	1431.91
B2A18		4282.44
B2A19	4.02	1257.71
B2A20	3.80	1284.42
B2A21	4.69	1448.85
B2A22	6.37	1569.34
B3A01	2.30	986.40
B3A02	1.71	945.94
B3A03	4.50	1304.00
B3A04	3.58	706.72
B3A05	4.69	1234.96
B3A06	3.51	1042.32
B3A07	4.29	1274.64
B3A08	2.84	1042.03
B3A09	4.13	1196.25
B3A11	3.44	1004.40
B3A12	6.33	1306.05
B3A13	3.21	1091.04
B3A14	3.33	1273.86
B3A16	5.99	1359.26
B3A17	2.96	1041.19
B3A19	2.83	1023.81
B3A20	2.57	1005.64
B3A21	2.72	1068.55
B3A22	3.33	1284.63
B3A23	2.68	1369.23
B3A24	3.62	1297.87
B4A01	3.50	1207.52
B4A02		
B4A04	4.32	1208.07
B4A06	3.44	921.72
B4A07	4.49	1072.38
B4A08	2.55	955.57
B4A09	2.41	895.14
B4A10	3.48	1377.37
B4A11	3.19	1167.78
B4A12	2.38	852.41

B4A13	1.81	1008.13
B4A14		
B4A15	3.89	1511.37
B4A16	3.15	1439.37
B4A17	2.57	1130.01
B4A18	5.13	1428.55
B4A20	2.85	1158.55
B4A21	3.06	1010.41
B4A22	2.64	1107.95
Date(s)	2013-09-17	
**Plot**		
B1A01	3.97	1147.68
B1A02	3.50	865.15
B1A03	3.63	908.42
B1A04	7.50	1010.73
B1A05	2.70	572.03
B1A06	4.02	981.85
B1A07	4.63	1000.60
B1A08	2.83	732.41
B1A11	3.87	1016.60
B1A12	4.38	1017.33
B1A13	2.63	649.09
B1A14		
B1A15	2.72	696.64
B1A16		986.56
B1A17	3.48	775.60
B1A18	2.60	
B1A19	4.16	1068.63
B1A20	3.95	1056.64
B1A21	3.10	985.04
B1A22	4.56	1153.17
B2A01		
B2A02	4.37	894.57
B2A03	4.46	1107.48
B2A04	2.02	512.20
B2A05	2.13	571.08
B2A06	3.21	999.59
B2A08	2.50	907.10
B2A09	3.86	1107.18
B2A10	5.74	1562.30
B2A12		
B2A13	3.79	812.98
B2A14	4.45	1275.09
B2A15	3.41	769.95
B2A16		
B2A17	4.76	1343.24
B2A18	4.84	1456.61
B2A19	4.38	1215.17
B2A20	6.20	1316.44
B2A21	4.37	1364.72

B2A22	5.28	1373.46
B3A01	2.99	960.21
B3A02	2.24	773.73
B3A03	3.20	1135.43
B3A04	3.25	911.57
B3A05	3.19	926.20
B3A06	3.33	1001.11
B3A07	2.90	1137.70
B3A08		
B3A09	3.93	1222.63
B3A11	2.90	792.64
B3A12	2.26	786.17
B3A13	3.29	923.13
B3A14	3.67	1186.91
B3A16	2.65	848.89
B3A17	2.65	848.89
B3A19	3.61	996.48
B3A20	3.00	948.56
B3A21	6.48	955.37
B3A22	4.86	1476.15
B3A23	4.60	1409.03
B3A24	3.63	1110.62
B4A01	3.98	1341.10
B4A02	5.25	1228.54
B4A04	5.32	1139.88
B4A06	3.80	1245.93
B4A07		
B4A08	4.60	1085.46
B4A09	3.72	1050.68
B4A10	3.58	1188.64
B4A11		
B4A12	2.87	835.72
B4A13	2.06	680.88
B4A14	3.67	1285.16
B4A15	3.04	1051.66
B4A16	3.29	1102.07
B4A17	2.57	862.63
B4A18	5.10	1533.96
B4A20	3.73	1270.68
B4A21	3.06	891.35
B4A22	3.69	978.93
