# Supplementary material for: Flood-Induced Changes in Soil Microbial Functions as Modified by Plant Diversity
Source: PLoS One. 2016 Nov 21;11(11):e0166349. doi: 10.1371/journal.pone.0166349 (PMC5117659; doi:10.1371/journal.pone.0166349)
Supplement: S2 Table — (PDF) [file pone.0166349.s002.pdf]

**Dataset**	Microbial growth after addition of Glucose	Microbial growth after the addition of Glucose and Nitrogen	Microbial growth after the addition of Glucose and Phosphorus	Microbial growth after the addition of Glucose, Nitrogen and Phosphorus
Unit	Slopes of microbial growth			
Type	raw	raw	raw	raw
Date(s)	2013-05-16;			
**Plot**				
B1A01	-0.01	0.01	0.02	0.08
B1A02	0.03	0.07	0.04	0.11
B1A03	0.02	0.03	0.03	0.12
B1A04	0.04	0.08	0.06	0.11
B1A05	0.04	0.08	0.08	0.15
B1A06	0.05	0.08	0.02	0.11
B1A07	0.01	0.08	0.04	0.13
B1A08	0.04	0.05	0.05	0.11
B1A11	0	0.06	0.01	0.09
B1A12	0.01	0.01	0.04	0.11
B1A13	0.03	0.03	0.05	0.1
B1A14	0	0.06	0.02	0.13
B1A15	0.03	0.09	0.04	0.11
B1A16	0.03	0.09	0.03	0.12
B1A17	0.04	0.1	0.05	0.13
B1A18	0.03	0.09	0.04	0.09
B1A19	0.05	0.09	0.08	0.11
B1A20	0	0.04	0.02	0.07
B1A21	0.02	0.06	0.03	0.09
B1A22	-0.02	0.03	-0.02	
B2A01	0.02	0.04	0.03	0.1
B2A02	0.01	0.03	0.01	0.12
B2A03	0.01	0.03	0.02	0.07
B2A04	0.05	0.09	0.06	0.12
B2A05	0.04	0.1	0.06	0.1
B2A06	0.03	0.07		
B2A08	0.04	0.08	0.04	0.11
B2A09	0.01	0.06	0.02	0.08
B2A10	0	0.04	-0.02	0.07
B2A12	0.03	0.05	0.03	0.09
B2A13	0.02	0.03	0.05	0.12
B2A14	0.02	0.06	0.05	0.13
B2A15	0.04	0.05	0.05	0.13
B2A16	0.03	0.08	0.02	
B2A17	0.02	0.06	0.02	0.08
B2A18	0.02	0.1	0.03	0.1
B2A19	0.03	0.08		0.11
B2A20	0.02	0.06	0.04	0.09
B2A21	0.01	0.04	0.02	0.07
B2A22	0.01	0.02	0.03	0.09
B3A01	0.01	0.02	0.02	0.1

B3A02	0.02	0.05	0.04	0.11
B3A03	0	0.01	0.02	0.08
B3A04	0.01	0.07	0	0.1
B3A05	-0.01	0.05	0.01	
B3A06	0.01	0.09	0.04	0.1
B3A07	0.02	0.09	0.06	0.12
B3A08	0.06	0.1	0.06	0.13
B3A09	-0.02	0.07	-0.02	0.1
B3A11	-0.02	0.07	-0.02	0.1
B3A12	0.06	0.08	0.06	0.11
B3A13	0.02		0.02	0.09
B3A14	-0.03	0.01	-0.02	0.06
B3A16	0.02	0.03	0.02	0.08
B3A17	0.04	0.07		
B3A19	0.04	0.09	0.04	0.1
B3A20	0.03	0.06	0.03	0.08
B3A21	0.04	0.06	0.05	0.09
B3A22	0.01	0.04	0.01	
B3A23	0.01	0.04	0.02	0.06
B3A24	0.01	0.06	0.03	0.07
B4A01	0	0.01	0.01	0.09
B4A02	-0.02	0.05	-0.02	0.09
B4A04	-0.02	0.03	-0.02	0.07
B4A06	0.01	0.06	0.03	0.09
B4A07	0.01	0.01	0.04	0.07
B4A08	-0.03	0.08	-0.02	0.1
B4A09	0.01	0.04	0.05	0.09
B4A10	-0.02	0.05	-0.02	0.09
B4A11	0.02	0.02	0.04	0.1
B4A12	0.04	0.08	0.05	0.14
B4A13	0.03	0.06	0.05	0.1
B4A14	0.04	0.07	0.04	0.11
B4A15	0.02	0.04	0.04	0.08
B4A16	0	0.07	0.01	0.08
B4A17	0.04	0.08	0.04	0.1
B4A18	0.02	0.04	0.02	0.07
B4A20	0.02	0.05		0.1
B4A21	0.03	0.04	0.06	
B4A22	0.01	0.07	0	
Date(s)	2013-07-01;			
Plot				
B1A01	0.01	0.02	0.04	0.1
B1A02	0.03	0.07	0.05	0.1
B1A03	0.03	0.05	0.05	0.1
B1A04	0.03	0.05	0.03	0.09
B1A05	0.03	0.08	0.04	0.12
B1A06	0.02	0.04	0.03	0.08
B1A07	0.02	0.07	0.03	0.1
B1A08	0.02	0.05	0.03	0.09
B1A11	0.02	0.04	0.03	0.08

B1A12	0.02	0.02	0.04	0.09
B1A13	0.05	0.05	0.08	0.12
B1A14	0.04	0.07	0.05	0.08
B1A15	0.05	0.08	0.04	0.11
B1A16	0.03	0.08	0.05	0.11
B1A17	0.03	0.08	0.05	0.11
B1A18	0.02	0.08	0.03	0.1
B1A19	0.01	0.07	0.02	0.12
B1A20	0.01	0.03	0.04	0.08
B1A21	0.03	0.05	0.03	0.09
B1A22	0.02	0.03	0.03	0.08
B2A01	0.01	0.02	0.03	0.06
B2A02	0.03	0.04	0.04	0.09
B2A03	0.02	0.04	0.04	0.08
B2A04	0.06	0.05	0.06	0.09
B2A05	0.04	0.07	0.05	0.1
B2A06	0.03	0.04	0.04	0.07
B2A08	0.01	0.07	0.02	0.08
B2A09	0.01	0.03	0.02	0.04
B2A10	0.02	0.04	0.03	0.06
B2A12	0.04	0.06	0.06	0.11
B2A13	0.04	0.05	0.07	0.1
B2A14	0.03	0.04	0.02	0.08
B2A15	0.07	0.07	0.05	0.11
B2A16	0.03	0.05	0.04	0.07
B2A17	0.04	0.05	0.05	0.08
B2A18	0.04	0.04	0.04	0.07
B2A19	0.02	0.05	0.03	0.12
B2A20	0.02	0.05	0.03	0.11
B2A21	0.02	0.03	0.02	0.13
B2A22	0.03	0.04	0.03	0.07
B3A01	0.03	0.03	0.06	0.1
B3A02	0.04	0.04	0.06	0.1
B3A03	0.02	0.02	0.04	0.07
B3A04	0.04	0.07	0.06	0.1
B3A05	0.03	0.05	0.05	0.08
B3A06	0.04	0.09	0.05	0.22
B3A07	0.03	0.05	0.04	0.16
B3A08	0.03	0.08	0.04	0.19
B3A09	0	0.06	0.02	0.08
B3A11	0	0.06	0.02	0.09
B3A12	0.03	0.06	0.04	0.08
B3A13	0.02	0.03	0.03	0.08
B3A14	0.02	0.03	0.03	0.05
B3A16	0.04	0.05	0.04	0.07
B3A17	0.05	0.07	0.06	0.1
B3A19	0.04	0.07	0.05	0.1
B3A20	0.03	0.05	0.04	0.09
B3A21	0.03	0.05	0.05	0.09
B3A22	0	0.04	0.02	0.06

B3A23	-0.01	0.02	0.02	0.07
B3A24	0.02	0.05	0.04	0.05
B4A01	0	0.01	0.02	0.05
B4A02	0.03	0.04	0.05	0.09
B4A04	0.01	0.02	0.03	0.07
B4A06	0.04	0.06	0.04	0.08
B4A07	0.01	0.02	0.05	0.08
B4A08	0.03	0.04	0.04	0.1
B4A09	0.01	0.01	0.03	0.08
B4A10	-0.02	0.04	-0.02	0.09
B4A11	0.02	0.05	0.04	0.11
B4A12	0.05	0.07	0.06	0.12
B4A13	-0.01	0.05	0.02	0.1
B4A14	0.03	0.06	0.04	0.09
B4A15	0	0.03	0.02	0.09
B4A16	0.02	0.04	0.03	0.08
B4A17	0	0.02	0.02	0.09
B4A18	0.01		-0.02	0.07
B4A20	0.01		0.02	0.1
B4A21	0.02		0.03	0.1
B4A22	0		0.01	0.09
Date(s)	2013-09-17;			
Plot				
B1A01	0.01	0.01	0.02	0.08
B1A02	0.02	0.06	0.02	0.1
B1A03	0.03	0.05	0.03	0.1
B1A04	0.03	0.05	0.04	0.09
B1A05	0.06	0.09	0.06	0.11
B1A06	0.01	0.05	0.01	0.08
B1A07	0.02	0.07	0.02	0.1
B1A08	0.04	0.06	0.04	0.1
B1A11	0.01	0.04	0.01	0.07
B1A12	0.02	0.02	0.03	0.08
B1A13	0.04	0.05	0.04	0.11
B1A14	-0.02	0.04	0	0.07
B1A15	0.04	0.08	0.05	0.11
B1A16	0.04	0.07	0.04	0.09
B1A17	0.03	0.07	0.03	0.1
B1A18	0.03	0.07	0.03	0.1
B1A19	0	0.06	-0.02	0.07
B1A20	0.01	0.03	0.01	0.06
B1A21	0.02	0.04	0.02	0.07
B1A22	0.01	0.02	0.03	0.06
B2A01	0.01	0.02	0.02	0.08
B2A02	0.02	0.05	-0.03	0.1
B2A03	0.01	0.03	0.01	0.06
B2A04	0.05	0.09	0.04	0.13
B2A05	0.04	0.08	0.03	0.11
B2A06	0.02	0.04	0.01	0.06
B2A08	0.01	0.05	0.01	0.08

B2A09	0.01	0.03	0.02	0.04
B2A10	-0.02	0.03	-0.02	0.06
B2A12	0.02	0.05	0.03	0.1
B2A13	0.03	0.04	0.02	0.1
B2A14	0.01	0.04	0.02	0.06
B2A15	0.05	0.08	0.05	0.13
B2A16	0.03	0.04	0.03	0.04
B2A17	0.02	0.05	0.02	0.06
B2A18	0.01	0.04	0.01	0.05
B2A19	0.01	0.04	0.01	0.06
B2A20	0.03	0.05	0.03	0.07
B2A21	0.01	0.03	0.01	0.06
B2A22	0.02	0.03	0.03	0.05
B3A01	0.02	0.02	0.03	0.08
B3A02	0.03	0.03	0.04	0.1
B3A03	0.03	0.03	0.02	0.07
B3A04	0.03	0.07	0.03	0.08
B3A05	0.02	0.05	0.02	0.07
B3A06	0.02	0.05	0.02	0.08
B3A07	0.03	0.05	0.02	0.05
B3A08	0.04	0.08	0.03	0.11
B3A09	0.02	0.06	0.01	0.08
B3A11	-0.01	0.05	-0.02	0.08
B3A12	0.05	0.08	0.07	0.09
B3A13	-0.01	0.03	-0.02	0.09
B3A14	0.02	0.02	0.02	0.07
B3A16	0.03	0.04	0.03	0.07
B3A17	0.05	0.07	0.04	0.11
B3A19	0.03	0.07	0.03	0.09
B3A20	0.03	0.06	0.02	0.09
B3A21	0.04	0.05	0.04	0.09
B3A22	0.02	0.05	0.02	0.05
B3A23	0.02	0.08	0.02	0.06
B3A24	0.04	0.06	0.02	0.07
B4A01	-0.02	0.01	-0.02	0.07
B4A02	0	0.04	0.01	0.07
B4A04	0.01	0.02	-0.02	0.07
B4A06	0.02	0.05	0.02	0.08
B4A07	0.02	0.02	0.03	0.08
B4A08	-0.01	0.03	0.01	0.1
B4A09	0.02	0.02	0.04	0.08
B4A10	-0.02	0.04	-0.02	0.09
B4A11	0.02	0.05	0.02	0.09
B4A12	0.05	0.07	0.05	0.1
B4A13	0.03	0.06	0.03	0.13
B4A14	0.01	0.05	0.02	0.09
B4A15	0.03	0.04	0.04	0.09
B4A16	-0.02	0.04	-0.01	0.12
B4A17	0.04	0.07	0.04	0.1
B4A18	0.02	0.02	0.02	0.09

B4A20	0.03	0.02	0.03	0.08
B4A21	0.02	0.02	0.03	0.09
B4A22	0.02	0.06	0.01	0.08
