# Supplementary material for: Flood-Induced Changes in Soil Microbial Functions as Modified by Plant Diversity
Source: PLoS One. 2016 Nov 21;11(11):e0166349. doi: 10.1371/journal.pone.0166349 (PMC5117659; doi:10.1371/journal.pone.0166349)
Supplement: S3 Table — (PDF) [file pone.0166349.s003.pdf]

Dataset

Enzyme activity of	Phenol oxidase	Peroxidase	Arginine protease	Glucosaminidase	Glucoronidase
Type	raw	raw	raw	raw	raw
Unit	nmol g-1 h-1	nmol g-1 h-1	nmol g-1 h-1	nmol g-1 h-1	nmol g-1 h-1
Date(s)	2013-05-16;	2013-05-16;	2013-05-16;	2013-05-16;	2013-05-16;
**Plot**					
B1A01	0.04	0.05	24659.58	721.10	76.49
B1A02	0.04	0.05	21160.03	166.20	-24.01
B1A03	0.04	0.06	21268.80	-8.67	72.34
B1A04	0.04	0.06	15413.54	293.09	31.39
B1A05	0.04	0.04			
B1A06	0.05	0.05	23809.17	418.21	67.50
B1A07	0.04	0.06	16926.45	155.77	-81.71
B1A08	0.04	0.05	13528.47	202.96	15.21
B1A11	0.04	0.06	26828.39	379.57	141.38
B1A12	0.04	0.06	17868.46	53.99	19.05
B1A13	0.05	0.05	14205.15	200.04	-35.33
B1A14	0.04	0.05	21616.82	52.05	-17.89
B1A15	0.04	0.06	13492.09	217.77	-33.40
B1A16	0.05	0.06	18244.57	90.47	23.63
B1A17	0.05	0.06	11961.71	187.22	-45.01
B1A18	0.05	0.06	15963.27	191.54	60.51
B1A19	0.05	0.05	21155.58	363.17	42.01
B1A20	0.05	0.05	22536.86	485.52	142.89
B1A21	0.04	0.06	17573.44	376.88	26.67
B1A22	0.04	0.06	28219.75	227.24	-1.44
B2A01	0.05	0.05	20225.57	338.38	343.39
B2A02	0.03	0.05	19809.17	398.69	352.71
B2A03	0.05	0.06	25679.98	455.61	400.05
B2A04	0.04	0.06	14368.84	314.24	240.98
B2A05	0.03	0.06	14313.09	230.73	319.44
B2A06	0.02	0.05	25679.98	529.17	400.05
B2A08	0.03	0.06	14782.08	440.98	418.39
B2A09	0.04	0.05	18698.95	484.21	662.09
B2A10	0.03	0.06	31255.20	628.76	541.80
B2A12	0.04	0.06	26744.38	305.30	437.85
B2A13	0.02	0.05	26744.38	156.49	437.85
B2A14	0.02	0.06	10630.89	496.88	199.07
B2A15	0.01	0.05	10630.89	191.01	199.07
B2A16			15740.73	200.56	222.55
B2A17			31255.20	353.31	541.80
B2A18			14368.84	485.61	240.98
B2A19			17194.94	482.17	95.21
B2A20			20449.80	310.03	503.86
B2A21			20449.80	630.21	503.86
B2A22			14313.09	728.56	319.44
B3A01			10328.46	346.27	369.74
B3A02			13822.99	286.53	453.97
B3A03			14349.67	303.78	390.14

B3A04			13822.73	640.99	418.03
B3A05			22831.18	499.08	667.28
B3A06			12449.94	348.32	509.42
B3A07			7670.46	350.01	260.73
B3A08			13078.88	397.97	508.83
B3A09			13691.05	325.76	475.26
B3A11			19748.96	630.81	644.52
B3A12			10700.30	192.63	310.43
B3A13			16483.43	564.08	500.54
B3A14			21168.45	768.56	676.68
B3A16			16078.61	844.37	622.77
B3A17			11300.06	219.73	395.03
B3A19			13746.42	479.00	561.58
B3A20			15231.92	467.26	529.39
B3A21			12285.36	375.37	268.82
B3A22			17845.89	480.91	719.03
B3A23			29244.63	537.73	676.40
B3A24			20409.57	654.87	546.20
B4A01			18758.62	1054.25	800.61
B4A02			21338.42	798.68	758.79
B4A04			15133.89	905.54	699.60
B4A06			18816.55	-46.50	880.94
B4A07			17825.14	857.58	689.78
B4A08			14968.93	630.60	503.39
B4A09			12609.25	830.41	679.67
B4A10			13502.43	1069.04	916.01
B4A11			15830.45	751.20	736.57
B4A12			13129.30	519.99	454.36
B4A13			12018.55	388.72	507.84
B4A14			13862.46	539.77	710.39
B4A15			13805.02	501.09	904.94
B4A16			18452.70	540.30	754.67
B4A17			10650.09	751.35	539.84
B4A18			17496.58	1030.02	988.37
B4A20			14409.27	708.16	741.77
B4A21			11224.26	369.21	421.57
B4A22			12355.05	717.57	843.17
Date(s)	2013-07-01;	2013-07-01;	2013-07-01;	2013-07-01;	2013-07-01;
Plot					
B1A01	0.03	0.05	50040.39	1481.78	154.27
B1A02			39557.78	275.67	207.40
B1A03	0.03	0.05	35495.94	2165.28	202.38
B1A04	0.03	0.05	30199.96	1114.57	198.39
B1A05	0.04	0.05	31269.72	960.07	87.30
B1A06			37115.49	893.30	236.24
B1A07	0.03	0.05	36540.16	588.35	212.41
B1A08	0.03	0.05	24512.31	1135.41	131.57
B1A11	0.03	0.06	43154.34	1390.86	286.76
B1A12	0.03	0.06	37008.93	993.85	226.41
B1A13	0.03	0.06	25009.95	635.19	125.01

B1A14	0.04	0.06	35211.37	1273.17	211.19
B1A15	0.03	0.06	26093.99	475.69	114.85
B1A16	0.03	0.06	29629.06	672.83	55.48
B1A17	0.03	0.06	25461.55	802.38	165.52
B1A18	0.02	0.05	33779.21	514.98	169.15
B1A19	0.03	0.06	37880.03	827.83	106.70
B1A20	0.04	0.05	33882.62	1375.13	373.72
B1A21	0.06	0.05	32944.11	885.24	171.91
B1A22	0.06	0.06	39807.71	1836.17	337.83
B2A01	0.06	0.06	38526.29	1201.80	244.42
B2A02	0.07	0.06	42461.89	1568.97	482.23
B2A03			37505.74	1818.92	438.02
B2A04	0.06	0.06	33934.62	1190.36	265.81
B2A05	0.07	0.10	29813.10	1141.85	193.20
B2A06	0.06	0.11	36722.57	1221.08	469.21
B2A08	0.07	0.12	36026.71	788.19	351.98
B2A09	0.07	0.12	36722.57	942.87	469.21
B2A10	0.07	0.13	42461.89	1438.03	482.23
B2A12	0.06	0.11	41702.10	1223.34	374.49
B2A13	0.06	0.12	26889.50	1064.62	248.63
B2A14	0.07	0.13	44643.47	1487.49	270.91
B2A15	0.06	0.13	36026.71	1151.59	351.98
B2A16			41777.16	1678.44	428.11
B2A17	0.06	0.11	41702.10	1137.02	374.49
B2A18	0.07	0.12	39395.67	1979.73	514.75
B2A19	0.07	0.13	37206.71	1056.12	501.10
B2A20	0.07	0.12	41777.16	1676.81	428.11
B2A21	0.07	0.11	37505.74	1532.71	438.02
B2A22	0.07	0.13	44643.47	1542.65	270.91
B3A01			26002.24	943.85	344.66
B3A02			22513.48	1162.09	437.29
B3A03			46165.09	2594.30	524.24
B3A04			30333.07	1228.64	368.13
B3A05			37449.14	1917.70	437.31
B3A06			33582.57	1240.35	471.13
B3A07			31516.38	1817.48	591.75
B3A08			32741.60	1278.43	497.79
B3A09			40825.65	1665.38	506.88
B3A11			38454.51	1628.30	537.62
B3A12			30064.23	1407.20	439.69
B3A13			39206.30	2063.96	545.73
B3A14			34731.06	2302.69	584.30
B3A16			33065.89	3102.65	571.26
B3A17			31136.50	1152.06	631.62
B3A19			27639.81	1168.50	422.47
B3A20			26242.67	2227.34	529.66
B3A21			29397.40	1837.83	585.28
B3A22			41535.41	2209.44	734.92
B3A23			44802.71	2282.87	601.73
B3A24			44999.10	4158.09	641.63

B4A01			44438.71	2190.32	752.93
B4A02			33936.21	1514.17	470.10
B4A04			32007.96	1354.48	532.41
B4A06			32333.41	1457.52	517.49
B4A07			41610.36	1604.47	765.81
B4A08			36574.35	1348.45	429.94
B4A09			27513.99	1898.67	601.65
B4A10			41332.27	1770.80	626.64
B4A11			44203.22	2190.53	578.74
B4A12			29021.94	1091.97	461.03
B4A13			32735.10	2131.44	362.12
B4A14			40918.22	931.47	601.05
B4A15			40905.16	2813.64	605.42
B4A16			32091.48	1367.43	708.48
B4A17			33278.16	1658.86	757.32
B4A18			37955.60	2033.92	646.50
B4A20			32001.22	2256.40	453.64
B4A21			29058.73	2254.73	478.10
B4A22			30522.54	1849.95	467.47

Date	2013-09-17;	2013-09-17;	2013-09-17;	2013-09-17;	2013-09-17;
Plot					
B1A01	0.07	0.11	10665800.97	339154.47	92700.17
B1A02	0.07	0.12	7646054.45	341833.40	51274.58
B1A03			8998036.22	279382.27	80168.91
B1A04	0.07	0.12	8051363.45	115362.50	30280.83
B1A05	0.05	0.07	5046012.92	138074.50	46818.09
B1A06	0.06	0.08	11198616.20	170627.40	97659.75
B1A07			6982844.93	177894.03	74806.68
B1A08			6291016.42	172242.57	64127.01
B1A11	0.03	0.08	12927716.73	333678.05	140618.64
B1A12	0.04	0.07	7942389.09	175655.33	89601.14
B1A13	0.05	0.07	6509442.41	154773.80	50498.58
B1A14			9567242.59	412463.64	85383.41
B1A15			5942032.97	75052.85	63939.76
B1A16	0.05	0.08	8126882.38	177171.65	107000.41
B1A17	0.05	0.06	6389824.99	146727.29	83740.33
B1A18	0.04	0.08	6733734.45	171156.03	74149.73
B1A19	0.06	0.07	9699145.30	130555.21	50325.60
B1A20	0.06	0.08	9738097.31	630885.44	144717.37
B1A21	0.05	0.08	8988438.47	289591.39	96555.91
B1A22	0.04	0.08	11086266.57	642761.45	136159.69
B2A01	0.05	0.06	9633085.58	228469.13	104927.77
B2A02	0.03	0.08	9411233.85	414086.97	95577.05
B2A03	0.05	0.08	11198425.94	285016.21	122612.83
B2A04	0.04	0.10	5631714.13	117287.61	59575.41
B2A05	0.04	0.10	5164766.29	178943.85	94822.12
B2A06	0.03	0.07	10834506.27	284366.59	156493.32
B2A08	0.03	0.10	5003700.54	198908.96	71042.91
B2A09	0.03	0.11	9570872.74	248893.15	239237.72
B2A10	0.04	0.09	11161731.61	367307.86	185160.93

B2A12	0.05	0.07	5312197.45	252760.12	40547.83
B2A13	0.03	0.09	5312197.45	91023.30	40547.83
B2A14	0.04	0.10	11386145.11	456825.21	135474.58
B2A15	0.08	0.14	5003700.54	141404.93	71042.91
B2A16			9063439.34	304920.15	137743.30
B2A17	0.08	0.15	5631714.13	258936.61	59575.41
B2A18	0.08	0.15	5164766.29	492433.41	94822.12
B2A19	0.09	0.15	10350433.25	246551.63	158175.63
B2A20	0.09	0.14	6339723.37	307466.04	142166.96
B2A21	0.06	0.14	9570872.74	172370.12	239237.72
B2A22	0.09	0.15	10834506.27	319880.68	156493.32
B3A01			8335410.49	224470.14	147198.08
B3A02			5319787.52	162697.15	123662.40
B3A03			7835553.16	184309.92	136107.02
B3A04			8584742.78	390183.75	96606.06
B3A05			9668213.27	328436.19	182336.18
B3A06			7918406.59	341074.47	157264.96
B3A07			7452494.03	313469.07	166440.51
B3A08			6749286.37	99438.57	110576.28
B3A09			8671611.72	270598.12	154151.40
B3A11			9761918.90	240629.26	113527.66
B3A13			8259144.31	279491.12	125668.46
B3A14			6339723.37	479458.77	142166.96
B3A16			8631512.04	481990.41	162023.28
B3A17			6923310.95	241027.42	140791.62
B3A19			6162047.38	320957.47	133512.14
B3A20			6490069.14	216098.58	133044.01
B3A21			6574326.74	215870.81	89961.42
B3A22			11159940.99	347311.15	209533.99
B3A23			13146087.85	312685.02	160741.71
B3A24			7684727.31	274749.66	141859.99
B4A01			11792993.01	531794.29	256037.08
B4A02			9184312.82	293127.84	144629.25
B4A04			8211559.65	265469.46	154377.06
B4A06			8336898.86	555473.53	167441.72
B4A07			7384782.30	167979.32	133179.05
B4A08			7858053.65	247533.15	133780.19
B4A09			7271661.51	465201.47	159125.74
B4A10			8384047.99	474918.60	166523.00
B4A11			8267321.83	472110.30	180840.58
B4A12			5204177.58	214835.16	144910.37
B4A13			5510446.99	193569.39	98876.37
B4A14			5977230.45	205380.96	128806.97
B4A15			6144202.33	289903.68	162433.80
B4A16			7998027.25	167176.21	206820.69
B4A17			4880259.45	260747.66	96511.45
B4A18			10149671.67	693304.84	221308.32
B4A20			7507546.33	374901.23	144517.03
B4A21			4940903.98	215225.61	95060.61
B4A22			7747582.53	321906.80	177869.05

Dataset

Enzyme activity of	Phosphatase	Galactosidase	Sulfatase	Tyrosin protease
Type	raw	raw	raw	raw
Unit	nmol g-1 h-1	nmol g-1 h-1	nmol g-1 h-1	nmol g-1 h-1
Date(s)	2013-05-16;	2013-05-16;	2013-05-16;	2013-05-16;
**Plot**				
B1A01	739.62	3614.80	364.96	11496.09
B1A02	56.96	896.95	225.29	10878.40
B1A03	233.96	952.24	272.98	11201.96
B1A04	524.85	2645.24	275.27	9275.86
B1A05				
B1A06	305.46	2061.32	434.35	11663.62
B1A07	79.30	996.88	223.71	10490.44
B1A08	375.88	1378.18	352.85	7364.52
B1A11	543.41	2110.11	516.88	12905.63
B1A12	251.16	911.97	263.25	12360.66
B1A13	277.90	1664.10	238.54	7804.91
B1A14	144.88	987.75	274.83	8360.04
B1A15	482.93	1926.74	214.36	8944.98
B1A16	80.09	747.63	302.04	10340.75
B1A17	218.33	1889.52	207.25	7454.52
B1A18	223.17	1475.48	306.73	10178.49
B1A19	487.84	2726.55	440.56	12890.92
B1A20	577.38	2328.43	656.03	10187.04
B1A21	386.23	2323.38	311.91	10376.36
B1A22	207.03	1397.73	360.44	15145.39
B2A01	420.81	2138.46	79.91	11064.59
B2A02	448.14	1802.51	177.78	11092.20
B2A03	533.60	2314.05	145.59	11300.29
B2A04	378.10	1586.86	86.08	9962.82
B2A05	350.68	1495.12	51.46	8952.21
B2A06	546.00	2433.81	145.59	11300.29
B2A08	465.63	2316.80	100.91	11321.87
B2A09	601.07	3247.65	80.09	13358.76
B2A10	709.97	3480.01	25.68	15019.26
B2A12	491.26	2438.44	23.34	13510.43
B2A13	284.02	1493.55	23.34	13510.43
B2A14	602.00	2331.02	47.91	8700.84
B2A15	407.57	1392.29	47.91	8700.84
B2A16	207.76	1541.46	25.92	9654.75
B2A17	558.59	2552.87	25.68	15019.26
B2A18	615.06	2509.08	86.08	9962.82
B2A19	531.69	2122.89	417.88	12543.61
B2A20	461.33	2106.32	88.47	13510.64
B2A21	930.13	3366.00	88.47	13510.64
B2A22	873.30	3326.71	51.46	8952.21
B3A01	1005.57	1245.45	347.34	11522.05
B3A02	444.37	2445.35	110.45	12218.59
B3A03	1115.00	1631.38	370.56	15404.48

B3A04	828.44	2401.96	144.22	11301.42
B3A05	765.28	2765.36	125.19	12322.30
B3A06	1433.82	1554.34	462.87	15126.06
B3A07	427.39	2327.00	83.04	6396.63
B3A08	560.81	2177.18	94.54	12278.89
B3A09	1083.24	1805.35	318.24	13124.59
B3A11	724.56	3206.76	134.13	18710.17
B3A12	952.43	1143.01	252.81	8968.69
B3A13	790.26	2617.93	53.66	9958.67
B3A14	880.27	3215.83	124.54	12764.88
B3A16	1111.59	3268.14	84.63	14462.39
B3A17	1206.26	1500.86	348.76	14535.49
B3A19	663.09	2437.76	108.96	14345.46
B3A20	518.44	2719.06	72.61	16018.75
B3A21	559.73	2003.19	85.99	9028.74
B3A22	1923.13	2309.42	570.49	18529.51
B3A23	765.28	2920.77	102.55	19367.60
B3A24	923.50	3024.68	86.78	11178.66
B4A01	1471.57	4509.22	69.29	11596.44
B4A02	1225.18	4192.82	230.73	15161.48
B4A04	1085.04	3675.15	115.64	12616.74
B4A06	386.59	-91.50	229.70	15500.07
B4A07	1294.84	3706.05	85.38	11900.27
B4A08	879.24	3276.13	111.58	8956.93
B4A09	1392.90	3233.26	120.27	10261.72
B4A10	1460.97	5329.89	134.87	11180.07
B4A11	1349.24	3649.56	169.16	11270.10
B4A12	671.62	2396.70	55.81	11038.36
B4A13	795.55	2425.85	145.36	9523.44
B4A14	1035.14	3407.83	166.68	14945.19
B4A15	918.07	2885.91	192.89	13030.48
B4A16	873.54	3372.08	80.42	11804.57
B4A17	764.96	2536.36	124.95	10603.50
B4A18	1346.54	4682.06	111.76	13591.26
B4A20	950.86	3109.63	190.77	12575.72
B4A21	747.12	2049.57	104.84	9739.91
B4A22	1224.96	4036.77	138.52	10285.72
2013-07-01;	2013-07-01;	2013-07-01;	2013-07-01;	2013-07-01;
Plot				
B1A01	2094.00	4795.88	-2.67	8767.21
B1A02	328.39	1339.55	214.41	8334.51
B1A03	2020.05	6186.71	204.96	7316.57
B1A04	1174.11	3811.93	131.25	6128.58
B1A05	1533.91	3662.67	47.25	5585.51
B1A06	1674.34	3606.48	180.40	8098.47
B1A07	851.29	2516.42	84.14	6316.50
B1A08	1251.47	3110.93	-0.36	5609.92
B1A11	2156.33	4274.40	182.13	10646.12
B1A12	1552.62	2858.45	63.87	8423.49
B1A13	1095.49	2741.03	4.11	7121.96

B1A14	2057.74	3655.58	116.42	6055.39
B1A15	185.74	2049.49	-15.92	5977.23
B1A16	1093.00	2582.38	-36.03	6805.06
B1A17	1239.49	3646.54	116.20	6549.62
B1A18	171.63	2300.92	24.91	7135.43
B1A19	1387.97	2747.41	56.02	9976.56
B1A20	1927.64	7050.49	183.83	8572.49
B1A21	917.66	3265.15	163.36	7785.69
B1A22	2398.59	4801.47	215.93	8154.28
B2A01	2073.27	3923.52	107.65	7710.78
B2A02	2400.35	4773.29	174.93	9397.57
B2A03	2191.95	4955.55	165.75	7972.35
B2A04	2028.47	3640.89	68.59	7751.92
B2A05	1433.88	3425.45	96.17	7549.91
B2A06	2570.13	4928.55	128.40	8756.69
B2A08	1388.38	3933.46	64.97	8940.57
B2A09	1771.85	4427.72	128.40	8756.69
B2A10	2292.05	4869.92	174.93	9397.57
B2A12	1959.53	4488.24	244.96	9298.74
B2A13	1263.74	2874.99	90.16	7760.25
B2A14	1782.75	4644.11	59.14	10190.68
B2A15	1633.14	2906.66	64.97	8940.57
B2A16	2601.87	6291.52	124.83	11879.54
B2A17	2379.13	4329.28	244.96	9298.74
B2A18	2125.82	4947.68	228.06	9588.46
B2A19	2464.46	5093.31	171.95	10055.14
B2A20	1710.27	5494.83	124.83	11879.54
B2A21	2533.73	5560.00	165.75	7972.35
B2A22	3067.99	5446.58	59.14	10190.68
B3A01	2954.12	4719.11	198.17	9229.83
B3A02	1868.97	4123.44	169.73	7712.60
B3A03	4975.82	7224.47	192.74	10821.96
B3A04	2965.65	4577.03	136.95	8425.92
B3A05	3643.29	7590.80	229.87	9137.71
B3A06	2831.92	5360.45	187.00	9602.00
B3A07	3086.01	5300.88	218.15	8986.73
B3A08	3576.75	6134.34	153.81	11497.00
B3A09	3321.13	5982.85	256.49	12080.95
B3A11	4086.72	4751.69	132.92	13607.68
B3A12	2358.69	4193.84	227.69	9013.22
B3A13	3165.77	6397.43	213.41	10068.04
B3A14	4913.33	7008.31	211.73	10573.70
B3A16	6624.56	7674.89	271.55	8460.72
B3A17	3701.24	5540.11	233.75	9628.02
B3A19	2610.73	4874.72	135.46	8484.44
B3A20	3878.82	7414.71	248.08	8436.22
B3A21	3191.23	8364.15	160.69	11141.62
B3A22	4263.20	9053.32	241.57	9794.04
B3A23	6574.92	7162.42	176.82	9471.72
B3A24	8113.03	9238.44	300.54	9776.43

B4A01	5599.93	7340.49	296.32	10631.83
B4A02	3265.08	4661.07	189.50	8035.43
B4A04	3145.36	4819.50	277.40	7273.19
B4A06	4975.98	5429.87	242.04	7692.06
B4A07	3303.92	4547.00	373.44	9588.26
B4A08	4806.05	7782.00	305.47	9633.38
B4A09	3649.76	5481.07	225.88	6579.91
B4A10	4148.13	6059.00	277.17	10180.29
B4A11	5067.74	8406.66	337.03	11836.09
B4A12	3302.51	4247.21	130.17	9484.33
B4A13	5284.03	8961.50	263.19	10582.11
B4A14	3149.79	4785.27	155.54	8965.20
B4A15	5671.37	10519.69	368.63	10785.90
B4A16	2708.53	5109.03	238.99	7860.29
B4A17	4116.44	5369.37	213.82	8425.84
B4A18	3760.19	5502.23	227.28	10287.72
B4A20	4423.60	7902.00	315.87	9972.76
B4A21	7844.44	8665.59	291.41	8790.94
B4A22	4873.60	9951.10	288.69	10179.66
2013-09-17;	2013-09-17;	2013-09-17;	2013-09-17;	2013-09-17;
Plot				
B1A01	396280.72	1195517.60	30333.72	2561866.76
B1A02	236351.82	873394.55	25448.92	1795316.40
B1A03	311555.96	758433.83	14326.41	1979110.70
B1A04	5486.62	627041.80	20421.24	1979253.15
B1A05	70203.65	695003.11	4456.67	1147331.02
B1A06	201558.26	866142.10	44617.42	2490995.12
B1A07	213352.94	657353.54	24674.40	2017256.82
B1A08	278454.83	599528.23	19822.49	1697518.92
B1A11	475766.09	1129545.69	34727.31	2872140.82
B1A12	406082.30	831370.30	23361.82	2340903.54
B1A13	124531.91	587824.12	18121.69	1775824.18
B1A14	475658.36	876661.81	43744.62	2376924.50
B1A15	94598.96	391666.41	16921.04	1680321.53
B1A16	481046.40	790069.14	38807.49	2513644.69
B1A17	220475.95	686077.37	13166.46	1782926.33
B1A18	349943.64	914575.99	27209.26	1999111.96
B1A19	119607.63	901004.33	21062.27	2355229.96
B1A20	568764.02	1360222.50	51468.06	2572591.03
B1A21	622895.40	941316.81	35116.20	2494216.77
B1A22	676473.79	1502418.51	34769.36	2613113.18
B2A01	358091.07	905703.92	32570.99	2457458.99
B2A02	511455.18	967684.54	50591.34	2388884.89
B2A03	386146.24	932811.68	56513.31	2689916.08
B2A04	163809.26	466729.21	23504.60	1916844.84
B2A05	162223.83	681247.54	36691.57	1636080.98
B2A06	401369.92	846230.00	48209.64	2884173.47
B2A08	438998.00	853383.48	37423.97	1695829.41
B2A09	537931.05	1316357.88	74644.97	2971111.68
B2A10	635575.39	1358386.96	60512.56	3112418.23

B2A12	502734.15	916770.00	-4609.42	1415280.23
B2A13	274824.14	588341.64	-4609.42	1415280.23
B2A14	346734.76	1173522.25	52178.85	3358623.27
B2A15	258742.53	587140.63	37423.97	1695829.41
B2A16	532798.02	1145396.35	70301.39	2546715.62
B2A17	852690.12	1117917.07	23504.60	1916844.84
B2A18	895093.44	1379398.20	36691.57	1636080.98
B2A19	591291.34	1216953.15	64369.07	3318419.95
B2A20	560683.38	954471.59	38166.89	2101564.59
B2A21	383438.95	891879.27	74644.97	2971111.68
B2A22	502046.93	1288516.67	48209.64	2884173.47
B3A01	426393.66	1020131.35	41098.44	3029542.85
B3A02	594563.34	764785.44	45199.50	2049134.91
B3A03	579258.54	945188.21	41404.04	2355079.81
B3A04	749319.45	971064.07	50429.34	2536147.83
B3A05	800029.98	1242880.73	84930.81	2788054.54
B3A06	749535.66	968609.99	65822.14	2454100.53
B3A07	813634.73	1109942.11	55924.16	2010600.98
B3A08	398487.79	812187.06	29977.02	2731135.20
B3A09	578663.67	961242.51	79100.53	2571459.10
B3A11	383030.88	812472.15	45423.12	3354849.94
B3A13	911992.81	1095523.20	53389.24	2715547.11
B3A14	1062934.44	1374157.62	38166.89	2101564.59
B3A16	1099819.54	1484150.79	55273.17	2639496.99
B3A17	864831.42	815038.96	55555.56	2893599.92
B3A19	834673.18	982766.87	44375.31	2361665.57
B3A20	570089.52	870804.08	49534.06	2362318.43
B3A21	441815.44	822047.57	16697.87	2811769.91
B3A22	937950.39	1244874.84	72303.62	3421225.07
B3A23	754888.02	1179811.09	34566.33	3760226.60
B3A24	959659.03	1080306.42	78428.98	1989763.94
B4A01	1674029.55	1665118.51	76242.14	3587954.97
B4A02	1374673.51	1360796.59	66281.00	3104582.31
B4A04	1096440.72	1019191.00	65120.68	2510750.30
B4A06	1385001.33	1668965.85	56289.02	2786171.66
B4A07	956785.04	582789.26	36276.69	3088493.95
B4A08	1079971.87	982011.57	58225.32	2728278.37
B4A09	1457478.63	1111457.06	47194.31	2198959.64
B4A10	1617175.42	1662596.66	57695.34	2763107.09
B4A11	1633262.11	1403469.68	71107.03	2721354.82
B4A12	1269210.42	739621.49	45200.39	1876842.86
B4A13	1285968.66	859289.78	51488.83	2006741.93
B4A14	1300259.71	1439915.49	46276.69	2109664.71
B4A15	1258536.54	1006552.83	50939.02	2501551.84
B4A16	748796.37	758105.29	50095.11	2450491.68
B4A17	1180947.49	894932.62	41595.90	1737680.90
B4A18	1646530.32	1509829.06	74017.40	3457888.86
B4A20	1294531.27	1225883.77	41402.68	2665279.80
B4A21	1329039.79	998707.78	35126.31	1736332.30
B4A22	1420553.52	1407254.78	72887.77	2403471.23
